# Efficient synthesis of stably adenylated DNA and RNA adapters for microRNA capture using T4 RNA ligase 1

**DOI:** 10.1038/srep15620

**Published:** 2015-10-26

**Authors:** Yunke Song, Kelvin J Liu, Tza-Huei Wang

**Affiliations:** 1 Biomedical Engineering Department, Johns Hopkins University, Baltimore, MD, 21218, USA; 2 Mechanical Engineering Department, Johns Hopkins University, Baltimore, MD, 21218, USA; 3 Circulomics Inc, Baltimore, MD, 21211, USA

## Abstract

MicroRNA profiling methods have become increasingly important due to the rapid rise of microRNA in both basic and translational sciences. A critical step in many microRNA profiling assays is adapter ligation using pre-adenylated adapters. While pre-adenylated adapters can be chemically or enzymatically prepared, enzymatic adenylation is preferred due to its ease and high yield. However, previously reported enzymatic methods either require tedious purification steps or use thermostable ligases that can generate side products during the subsequent ligation step. We have developed a highly efficient, template- and purification-free, adapter adenylation method using T4 RNA ligase 1. This method is capable of adenylating large amounts of adapter at ~100% efficiency and can efficiently adenylate both DNA and RNA bases. We find that the adenylation reaction speed can differ between DNA and RNA and between terminal nucleotides, leading to bias if reactions are not allowed to run to completion. We further find that the addition of high PEG levels can effectively suppress these differences.

MicroRNA are small non-coding RNA (17–24 nt) that regulate an estimated 60% of all post-transcriptional gene expression. Their rapidly growing impact across broad swaths of cell biology, molecular biology, developmental biology, clinical diagnostics, and therapeutics has driven the development many new methods to quantify their expression^1^,^2^. Due to their short length, many of microRNA assays begin with direct reverse transcription^3^,^4^,^5^, poly-A tailing^6^,^7^, or adapter oligonucleotide ligation^8^,^9^ to extend the length and add a defined sequence that renders them easier to handle. Among these methods, adapter ligation is arguably the most versatile and has been integrated into many microRNA expression profiling assays including RT-PCR^10^,^11^,^12^ and microarray^5^,^13^,^14^,^15^ as well as many sequencing library preparation methods^8^,^16^,^17^.

Ligases catalyze covalent bond formation between a 5′ phosphate (Ph) group donor and a 3′ hydroxyl (OH) acceptor. In microRNA adapter ligation, one of the predominant methods to minimize side product formation is to pre-adenylate the 5′ end (5′-App) of the adapter and perform the subsequent ligation in the absence of ATP or using a mutant ligase that lacks the adenylation domain (e.g. T4 RNA ligase 2, truncated). In this manner, ligation can only occur between the pre-adenylated 5′ adapter end and the 3′-OH of sample RNA. This method minimizes side product formation including microRNA circularization, microRNA polymerization, 5′ microRNA ligation, and the ligation of other nucleic acids to the microRNA 3′ end.

This method is only feasible if pre-adenylated adapters can be synthesized in large quantities and with high purity. Adapter pre-adenylation for microRNA detection was first performed by chemical synthesis, in which AMP was activated and adenosine 5′-phosphorimidazolidate was coupled to the 5′-phosphorylated oligonucleotide^18^,^19^. This method is complex and requires significant chemistry expertise as well as a final purification step. Subsequently, an enzymatic method using T4 DNA ligase was developed to achieve higher yield and to reduce process complexity^9^. As T4 DNA ligase requires a double stranded substrate, a template oligo had to be used which then required a subsequent denaturing and purification step to remove. With both methods, these purifications are challenging as they must be able to separate the adenylated product from the 1 nt shorter substrate. Recently, a template-independent enzymatic method has been reported using thermostable archaeal RNA ligase^20^. With this enzyme, adenylation readily occurs at 65 °C, but significant de-adenylation can occur at 25 °C, the optimum reaction temperature for many ligases used in the subsequent microRNA–adapter ligation (e.g. T4 RNA Ligase 2 truncated, T4 RNL2 trunc), thus reducing the total amount of usable adapter in the solution^21^. Furthermore, thermostable archaeal RNA ligase cannot be easily denatured and can lead to microRNA circularization when it is carried through from the adenylation step to the subsequent ligation step, hindering accurate microRNA quantification. This is an inevitable side effect as RNA circularization is a primary function of this enzyme^21^.

Herein, we report a one-step, purification-free method for efficient and stable adenylation of DNA and RNA-adapters using T4 RNA Ligase 1 (T4 RNL1). T4 RNL1 catalyzes phosphodiester bond formation between the phosphorylated 5′ terminal and the 3′ hydroxyl terminal in single stranded RNA^22^,^23^. First, the ligase first forms a covalent bond with ATP, generating a ligase-(lysyl-N)–AMP intermediate and releasing a pyrophosphate. Next, adenylated RNA (AppRNA) is formed as AMP is transferred from the ligase to the 5′-phosphate of RNA. As T4 RNL1 lacks the specificity to distinguish RNA from DNA, this reaction occurs for both RNA and DNA oligonucleotides. Finally, the ligase catalyzes 3′-5′ phosphodiester bond formation by attack of an RNA 3′-OH on the AppRNA or AppDNA, releasing AMP. If T4 RNL1 is mixed only with an oligonucleotide containing a 5′ Ph and blocked 3′-ddC end, the ligation reaction will terminate after step 2 because no 3′-OH acceptors are present in the reaction mixture. This leaves the adapter oligo with an activated 5′-App end group that can then be ligated in the absence of ATP. T4 RNL1 is not thermostable and can be easily heat-inactivated after adenylation, eliminating side product formation from enzyme carry-through. We explore the reaction parameters affecting T4 RNL1 adenylation efficiency and compare against archaeal RNA ligase. As a proof-of-feasibility, the pre-adenylated adapters were used to capture microRNA from total RNA samples in a highly efficient and unbiased manner using the protocol previously reported by our group^24^.

## Results

### RNA and DNA adenylation by T4 RNA ligase 1

**Figure 1:**
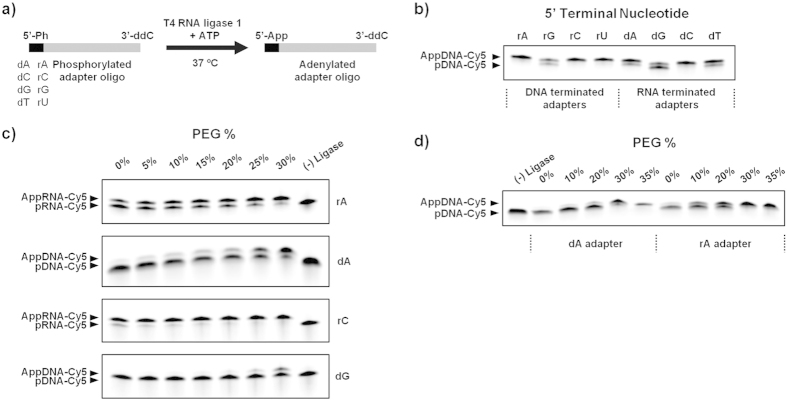
(**a**) Schematic illustration of the high efficiency, purification- and template-free, adapter oligonucleotide adenylation method using T4 RNA ligase 1. The 3′ end of the adapter oligo was blocked by –ddC modification to prevent circularization and concatemerization. The 5′ base (shown in black) was swapped between dA, dC, dG, dT, rA, rC, rG, and rU to test bias. (**b**) The adapter adenylation efficiency was investigated as a function of 5′ terminal nucleotide. The reaction conditions were modified to exaggerate differences in efficiency (10 μL volume, 100 units ligase per nanomole adapter, 0.1 nanomole adapter, 30% PEG, 1 hour incubation). The rC and dG adapters are the most and least efficiently adenylated, respectively. (**c**) The adapter adenylation efficiency was then measured as a function of PEG % for a few representative adapters. In all cases, efficiency monotonically increased with PEG %. (**d**) Comparison of adenylation efficiency of as a function of PEG % under standard reaction conditions using the rA and dA adapters. Both the dA and rA adapters are efficiently adenylated at 35% PEG.

A previously reported method used double stranded T4 DNA ligase to perform enzymatic adenylation^9^. We postulated that by switching to single stranded T4 RNA ligase 1, we would be able to eliminate the template and perform purification-free, adapter adenylation. As T4 RNL1 catalyses ligation between both single stranded DNA and RNA, we investigated whether this ligase would display any adenylation preference with regards to DNA vs. RNA or 5′ base composition (Fig. 1a). We compared the adenylation efficiency using a series of adapters that varied only at the 5′ base (dA, dC, dG, dT vs. rA, rC, rG, rU) (Supporting Table S1). Given that previous work has shown that polyethylene glycol (PEG) can have a dramatic effect on ligation efficiency and specificity^9^,^11^,^24^,^25^,^26^,^27^, we concurrently investigated whether adenylation bias could be suppressed by the addition of PEG. When the adenylated adapters are analyzed by polyacrylamide gel electrophoresis (PAGE), the adapter adenylation efficiency can be determined by comparing the intensity of the adenylated band against the 1 nt shorter phosphorylated band. Due to their short length, the 5′ terminal bases have a direct effect on the electrophoretic mobilities of their respective adapter oligos. To exaggerate the differences for investigational purposes, we added excess adapter into the reactions and reduced the enzyme levels. Under these conditions, the cytidine (rC and dC) and guanosine (rG and dG) terminated adapters were the most and the least efficiently adenylated, respectively (Fig. 1b). The adenosine (rA and dA) and thymidine/uridine (dT and rU) terminated adapters had intermediate efficiencies. When PEG % was varied, adenylation efficiency increased monotonically with PEG, but the critical PEG level at which adenylation efficiency maxed out was different depending on 5′ nucleotide composition (Fig. 1c). While the rC adapter was well adenylated at PEG levels as low as 5%, the dG adapter was not fully adenylated even at 30% PEG. Furthermore, as seen in Fig. 1d, at low PEG levels (<20%), the RNA terminated adapters were adenylated more efficiently than the DNA terminated adapters, but at high PEG levels (>35%), both RNA and DNA adapters were adenylated equally efficiently. Whereas ligation can sometimes be inhibited by very high PEG levels^24^, no such effect was seen for adenylation. Thus, 35% PEG was universally chosen for all subsequent adenylation reactions.

### Optimization of enzyme-to-adapter ratio, ATP concentration, adapter concentration and time

**Figure 2:**
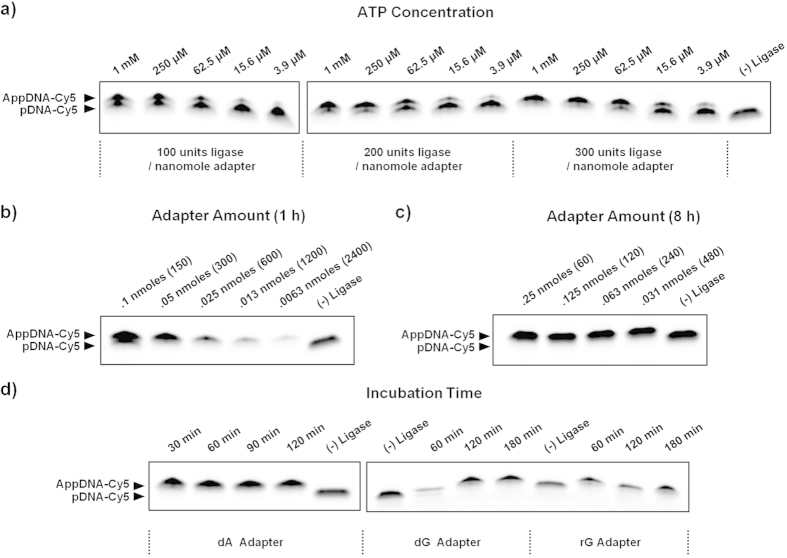
(**a**) The enzyme/substrate ratio and ATP concentration were varied in tandem to quantify their effect on adenylation efficiency. A combination of 300 units of ligase per nanomole adapter and 250 μM or more of ATP yields high adenylation efficiency. The reaction volume was 50 μL. Using these conditions, the adenylation efficiency was measured as a function of adapter concentration. (**b**) Up to 0.05 nanomoles of adapter could be fully adenylated in 1 hour (**c**) while 0.25 nanomoles could be adenylated with overnight incubation. The bracketed number indicates the enzyme substrate ratio (units per nanomole). (**d**) The adenylation efficiency was measured as a function of incubation time for a few representative adapter oligos. The dA adapter could be fully adenylated in <30 minutes while the dG and rG adapters required extended incubation of 120 minutes.

Next we examined the effects of enzyme/substrate ratio and ATP concentration on adenylation efficiency. Enzyme/substrate ratio should be optimized to balance cost and yield while ATP should be optimized to minimize carry-through into the subsequent ligation reaction. By varying enzyme and ATP input simultaneously, we find that highly efficient adapter adenylation can be obtained using 300 units of ligase per nanomole adapter and 250 μM ATP (Fig. 2a). We then determined the maximum amount of adapter that could be adenylated under these conditions by varying adapter input while holding enzyme and ATP levels constant (Fig. 2b). Up to 0.05 nanomoles of adapter could be fully adenylated within 1 hour, corresponding to an enzyme substrate ratio of 300 units of ligase per nanomole adapter. If over-night incubation is used instead, up to .25 nanomoles of adapter (10 μM concentration) could be fully adenylated under the same conditions (Fig. 2c). The ability to prepare adenylated adapters at such high concentrations facilitates downstream ligation reactions by preventing unnecessary dilution.

**Figure 3:**
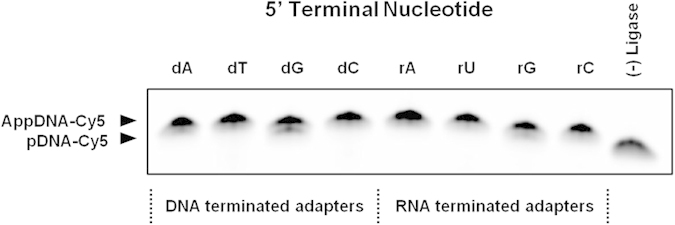
The adenylation efficiency of all 8 adapter versions was measured using the optimized protocol and 1 hour incubation time. All adapters were fully adenylated except the dG and rG adapter, which required additional time.

We then tested the effect of incubation time on adenylation efficiency and saw that the dA adapter could be completely adenylated in as little as 30 minutes (Fig. 2d). Based on the previous experiments, we performed adenylation on all 8 adapter variants using optimized conditions of 0.05 nanomole of adapter, 35% PEG, 1 mM ATP, and 300 units of T4 RNL1 per nanomole adapter in a 25 μL reaction volume. After incubating at 37 °C for 1 hour, the majority of adapters (Fig. 3) were perfectly adenylated except for the dG and rG adapters which required longer incubation times of 2 hours (Fig. 2d). The guanosine terminated adapters were adenylated significantly slower than the others and if not given sufficient time to run to completion, biases in adenylation efficiency and, potentially, subsequent ligation efficiency will result.

### Validation of the adenylated adapter

**Figure 4:**
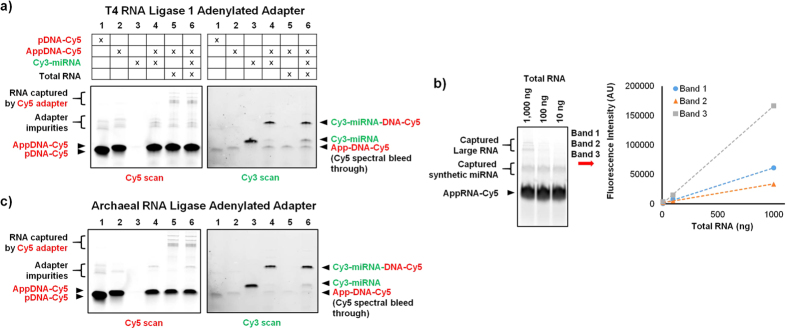
microRNA-adapter ligation was performed using adenylated adapters generated by either (a) T4 RNA ligase 1 or (c) archaeal RNA ligase. The adapters were labeled with Cy5 while the synthetic microRNA were labeled with Cy3. Lanes 1 and 2 show that both methods are capable of fully adenylating the adapters. Lanes 4 and 6 show that let-7a microRNA can be effectively ligated both in the absence and presence of total RNA background. Lane 5 shows that large RNA molecules within the total RNA are captured by both adapters. No de-adenylation is observed with either method. (**b**) The T4 RNA ligase 1 adenylated adapter was used to capture RNA from 10, 100, or 1000 ng of pancreatic tissue total RNA spiked with 0.01 picomoles of 6 synthetic microRNA. The three ligation products from the top are large RNA molecules intrinsic to the total RNA that have been captured by the adapter. As expected, they vary in linear proportion to the total RNA input. The band in the middle is the spiked microRNA captured by the adapter which remains constant across all three samples as expected. The large band at the bottom of the gel is free adenylated Cy5 adapter.

To test the functionality of the adenylated adapters, microRNA-adapter ligation was performed on synthetic let-7a microRNA and human total RNA samples (Fig. 4a). A 3′-Cy5 labeled dA adapter was adenylated using T4 RNL1 followed by heat-inactivation at 65 °C for 15 minutes. Ligation was then performed using T4 RNL2 trunc in the absence of ATP. T4 RNL2 trunc lacks the adenylation domain and can only perform ligation using a pre-adenylated adapter. The adapter was first used to capture synthetic let-7a microRNA that was labeled with a 5′-Cy3. The capture efficiency, defined as (Cy3 signal of captured let-7a band)/(the Cy3 signal of captured let-7a band + the Cy3 signal of free let-7a band), was 91%. The Cy5-labeled adenylated adapter was then used to capture RNA molecules from 100 ng of human pancreatic tissue total RNA. The RNA molecules were successfully captured as evidenced by the high molecular weight bands in the Cy5 gel scan. These are likely from the ligation of the adapter to rRNA. As another test, the Cy3-labeled let-7a microRNA was spiked into the total RNA samples and captured by the 3′-Cy5 labeled, adenylated adapter. In this case, the capture efficiency of the let-7a microRNA was 70%. At the same time, large RNA molecules within the total RNA were also captured by the adapter as evidenced by the ligation product bands in high molecular region of the Cy5 channel. This indicates that the pre-adenylated adapter, prepared with the proposed method, is capable of high efficiency capture of both microRNA and other RNA molecules within the total RNA sample.

As a final verification of adapter functionality, microRNA capture was performed on total RNA that was titrated from 1,000 ng down to 10 ng. 0.01 picomoles of six synthetic microRNA (let-7a, miR-16, miR-21, miR-26a, miR-29b and miR-34a) were spiked-in into each total RNA sample. As seen in Fig. 4b, the band intensity of ligation products in the high molecular weight region of the gel varied in direct proportion to the total RNA input. These bands represent total RNA-derived large RNA molecules captured by the adapter. This is in contrast to the spiked-in microRNA which were uniformly captured in all cases. This experiment demonstrates that the adenylated adapter is able to capture all RNA species proportionally to their presence within the total RNA, enabling accurate downstream quantification. We have also previously shown that these adenylated adapters can be used for low bias microRNA capture using T4 RNL2^24^.

### Ligation side product formation

To compare the current T4 RNL1 adenylation process against the previously reported thermostable archaeal RNA ligase adenylation process^20^, the dA adapter was adenylated using thermostable archaeal RNA ligase according to the manufacturer’s protocol, and the experiment from Fig. 4a was repeated. Using thermostable archaeal RNA ligase, the adapter was adenylated at high efficiency, and the RNA capture efficiency was comparable to that obtained using T4 RNL1 adenylated adapters (Fig. 4c)^24^. No substantial difference could be seen between the two methods in terms of either adenylation or microRNA capture efficiency. As previously described by multiple groups^20^,^21^, thermostable archaeal RNA ligase adenylates DNA at 65 °C but the reverse de-adenylation reaction can prevail at lower temperatures. As the subsequent ligation is often performed at 25 °C, it was expected that some de-adenylation would occur, as reported by Zhelovsky^20^. Despite this, we did not observe adapter de-adenylation with either method.

**Figure 5:**
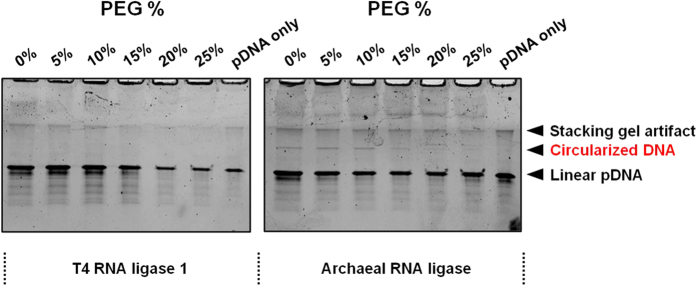
Adenylated adapters generated using either T4 RNA ligase 1 or archaeal RNA ligase were used for microRNA-adapter ligation of a mixture containing 80 nt let-7a precursor DNA molecules and 22 nt let-7a mature microRNA molecules. The amount of PEG in the reaction mixture was also varied. Circularized DNA ligation product is only generated using the archaeal RNA ligase adenylated adapters.

However, differences were observed in terms of side product generation, specifically circularization, with the archaeal RNA ligase-prepared adapter. As seen in Fig. 5, adenylated adapters, generated by either T4 RNL1 or archaeal RNA ligase, were mixed with an 80 nt, single stranded DNA oligo (let-7a precursor) and a 22 nt let-7a microRNA. Adapter ligation was then performed using T4 RNL2 trunc. The ligated samples were analyzed by denaturing PAGE and SYBR® Safe DNA Gel Stain. While the 80 nt DNA precursor was long enough to be efficiently stained and seen on the gel, the 22 nt let-7a microRNA and 19 nt adapter were not efficiently visualized on the gel. As T4 RNL2 trunc lacks the ability to adenylate, it can only ligate the 5′ adenylated adapter to the 3′ end of either the let-7a pre-cursor or the let-7a microRNA molecules. No other reactions can be catalyzed by T4 RNL2 trunc. Any remaining ligation reactions are the result of carry-through T4 RNL1 or archaeal RNA ligase activity. An extra band, resulting from let-7a precursor circularization, can be seen with the archaeal RNA ligase adapters but not the T4 RNL1 adapters. This is because T4 RNL1 is easily heat inactivated while archaeal RNA ligase is not. The amount of the circularized DNA decreased with higher PEG input but the circularization reaction could not be entirely quenched even at 25% PEG, the optimum PEG level for low bias microRNA-adapter ligation^24^.

## Discussion

We report a quick, simple, and robust way to synthesize pre-adenylated adapter oligos using T4 RNL1 that does not require a template or subsequent purification. De-adenylation is not seen to occur, and T4 RNL1 can be effectively heat-inactivated prior to the microRNA capture step, greatly reducing downstream side product formation. PEG, which is known to enhance the activity of T4 RNA ligases by molecular crowding^25^, can be used to suppress adenylation bias in the same manner as it suppresses ligation bias^24^. In contrast to microRNA–adapter ligation where high PEG levels can inhibit ligation efficiency, no reduction in adenylation efficiency was observed even at PEG levels as high as 35%. At 35% PEG, all of the adapters tested could be fully adenylated in 2 hours. In contrast to previous studies that reported inefficient DNA adenylation by T4 RNAL1^20^, we find that with sufficiently high PEG, T4 RNAL1 can obtain high yields and low adenylation bias. Much as we previously reported with ligation by T4 RNL2^24^, high PEG seems to relax enzyme stringency, enabling it to be less selective regarding substrates.

Although synthesis of adenylated adapters is essential for microRNA–adapter ligation, most of the methods proposed thus far are complex and involve tedious purification of the adenylated adapter. A purification-free, template-independent method has been previously demonstrated using thermostable archaeal RNA ligase^20^. However, this method has two potential drawbacks. First, adapter de-adenylation can occur, reducing the amount active adenylated adapter. Second, microRNA circularization can occur as a result of carry-through archaeal RNA ligase activity. To our best knowledge, the propensity of archaeal RNA ligase to generate microRNA circularization when performing adapter ligation has never been investigated. Our experiment using a 5′ phosphorylated DNA target implies that archaeal RNA ligase can indeed circularize nucleic acids as a result of carry-through activity. Unfortunately, due to limitations in fluorescent gel sensitivity, we are unable to see short RNA molecules labelled with SYBR green and end labelling using fluorophore would artificially block ligation. Yet it is reasonable to postulate that microRNA circularization may be even more pronounced as archaeal RNA ligase has higher activity for RNA than DNA. The amount of circularized microRNA could also be different depending on the ligation conditions. For instance, less circularized DNA was formed when high PEG levels were used. It is further expected that some microRNAs are more likely to be circularized than others due to sequence or secondary structure. Such circularization would introduce additional bias in microRNA expression profiling. In such cases, adapter adenylation by T4 RNL1 could be a safer alternative.

Finally, we have noticed substantial primary sequence bias in the adenylation process using T4 RNL1. However, we have not seen systematic primary sequence bias in the subsequent ligation step using T4 RNL2 trunc, K227Q^24^. Others have reported that the subsequent ligation step does not show primary sequence bias but does in fact show bias based on secondary structure effects^28^,^29^. An interesting study would be to examine whether the ultimate ligation bias occurs more predominantly due the initial donor adenylation step or the subsequent acceptor ligation step. However, the affect of adenylation bias would only be significant when pooled adapters are used.

## Methods

### Oligonucleotides

microRNA and adapter oligonucleotides were synthesized by Integrated DNA Technologies (Coralville, IA). MicroRNA sequences were obtained from miRBase v21 (www.mirbase.org). Each microRNA possessed Cy3 and –OH at their 5′ and 3′ termini, respectively. The adapter sequences were based on modified 3′ modban adapters^24^ and were labeled with phosphate and Cy5 at their 5′ and 3′ termini, respectively. All oligos were HPLC purified. The sequences are listed in Table S1.

### Adenylation Reaction.

Unless otherwise indicated, the adenylation reaction was performed using the optimized conditions of a 25 μL reaction volume containing 0.05 nanomole dA adapter, 1X T4 RNA Ligase Buffer (New England Biolabs, Ipswich, MA), 35% PEG, 1 mM ATP, and 300 units of T4 RNA Ligase 1 (New England Biolabs, Ipswich, MA) per nanomole adapter. Adenylation was performed by incubating at 37 °C for 1 hour, followed by heat-inactivation at 65 °C for 15 minutes. Alternatively, adapter pre-adenylation was performed using the 5′ DNA Adenylation Kit (New England Biolabs, Ipswich, MA) according to the manufacturer protocol.

microRNA-Adapter Ligation. microRNA was ligated to the pre-adenylated dA adapters using T4 RNA ligase 2 truncated K227Q (New England Biolabs, Ipswich, MA) according to the previously described protocol^24^. Pancreatic total RNA was commercially purchased from Life Technologies.

### PAGE Analysis

The samples were analyzed on precast 15% TBE-urea polyacrylamide gels (Bio-Rad, Hercules, CA). 5 μL of sample was mixed with 5 μL of loading buffer and heated for 5 minutes at 95 °C. The sample was then loaded into the gel and run for 20 minutes at 300 V. Oligonucleotide detection was performed using Cy3 or Cy5 fluorescent end labels or SYBR® Safe DNA Gel Stain (Life Technologies, Carlsbad, CA) when fluorescent end labelling was not possible.The separated gels were scanned using a Typhoon 9410 variable mode imager (GE Healthcare, Piscataway, NJ). Scans were performed at 488 nm excitation and 520 nm emission for SYBR, 532 nm excitation and 580 nm emission for Cy3, and 633 nm excitation and 670 nm emission for Cy5. The gel images were analyzed using ImageQuant (GE Healthcare, Piscataway, NJ) to obtain lane profiles and curve-fit using OriginPro to obtain band intensities.

## Additional Information

**How to cite this article**: Song, Y. *et al.* Efficient synthesis of stably adenylated DNA and RNA adapters for microRNA capture using T4 RNA ligase 1. *Sci. Rep.* **5**, 15620; doi: 10.1038/srep15620 (2015).

## Supplementary Material

Supplementary Information
