# Supplementary material for: Efficient synthesis of stably adenylated DNA and RNA adapters for microRNA capture using T4 RNA ligase 1
Source: Sci Rep. 2015 Oct 26;5:15620. doi: 10.1038/srep15620 (PMC4620478; doi:10.1038/srep15620)
Supplement: Supplementary Information [file srep15620-s1.pdf]

Efficient synthesis of stable adenylated DNA and RNA adapters for microRNA capture using T4 RNA ligase 1

Yunke Song[1], Kelvin J Liu[2,3,*] and Tza-Huei Wang[1,2,*]
[1] Biomedical Engineering Department, Johns Hopkins University, Baltimore, MD, 21218, USA
[2] Mechanical Engineering Department, Johns Hopkins University, Baltimore, MD, 21218, USA
[3] Circulomics Inc, Baltimore, MD, 21211, USA

*To whom correspondence should be addressed: Tel: +1 410 516 7086; Fax: +1 410 516 7254; Email: thwang@jhu.edu. Correspondence may also be addressed to: Tel: + 1 626 202 4825; Email: kliu@circulomics.com

Supplementary Data

**Name**	**Sequence 5' -> 3'**
dA	5' - /5Phos/ACTGTAGGCACCATCAATC/3Cy5Sp/ - 3'
dT	5' - /5Phos/TCTGTAGGCACCATCAATC/3Cy5Sp/ - 3'
dG	5' - /5Phos/GCTGTAGGCACCATCAATC/3Cy5Sp/ - 3'
dC	5' - /5Phos/CCTGTAGGCACCATCAATC/3Cy5Sp/ - 3'
rA	5' - /5Phos/rACTGTAGGCACCATCAATC/3Cy5Sp/ - 3'
rU	5' - /5Phos/rUCTGTAGGCACCATCAATC/3Cy5Sp/ - 3'
rG	5' - /5Phos/rGCTGTAGGCACCATCAATC/3Cy5Sp/ - 3'
rC	5' - /5Phos/rCCTGTAGGCACCATCAATC/3Cy5Sp/ - 3'
let-7a	/5Cy3/rUrGrArGrGrUrArGrUrArGrGrUrUrGrUrArUrArGrUrU
miR-16	/5Cy3/rUrArGrCrArGrCrArCrGrUrArArArUrArUrUrGrGrCrG
miR-21	/5Cy3/rUrArGrCrUrUrArUrCrArGrArCrUrGrArUrGrUrUrGrA
miR-26a	/5Cy3/rUrUrCrArArGrUrArArUrCrCrArGrGrArUrArGrGrCrU
miR-29b	/5Cy3/rUrArGrCrArCrCrArUrUrUrGrArArArUrCrArGrUrGrUrU
miR-34a	/5Cy3/rUrGrGrCrArGrUrGrUrCrUrUrArGrCrUrGrGrUrUrGrU
let-7a precursor DNA	/5Phos/TGGGATGAGGTAGTAGGTTGTATAGTTTTAGGGTCAC<br>ACCCACCACTGGGAGATAACTATACAATCTACTGTCTTTCCTA

Table S1. List of oligonucleotide sequences.

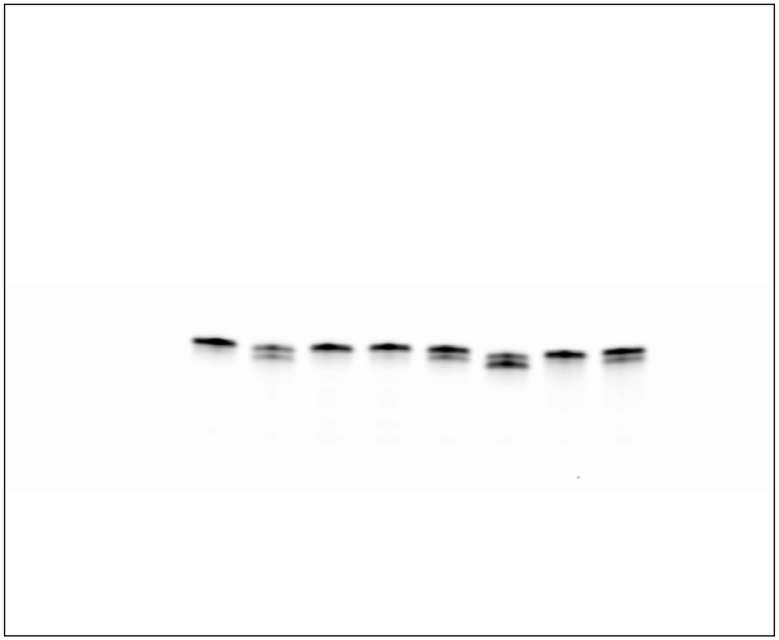

Supplemental Figure S1. Uncropped gel image of Figure 1b

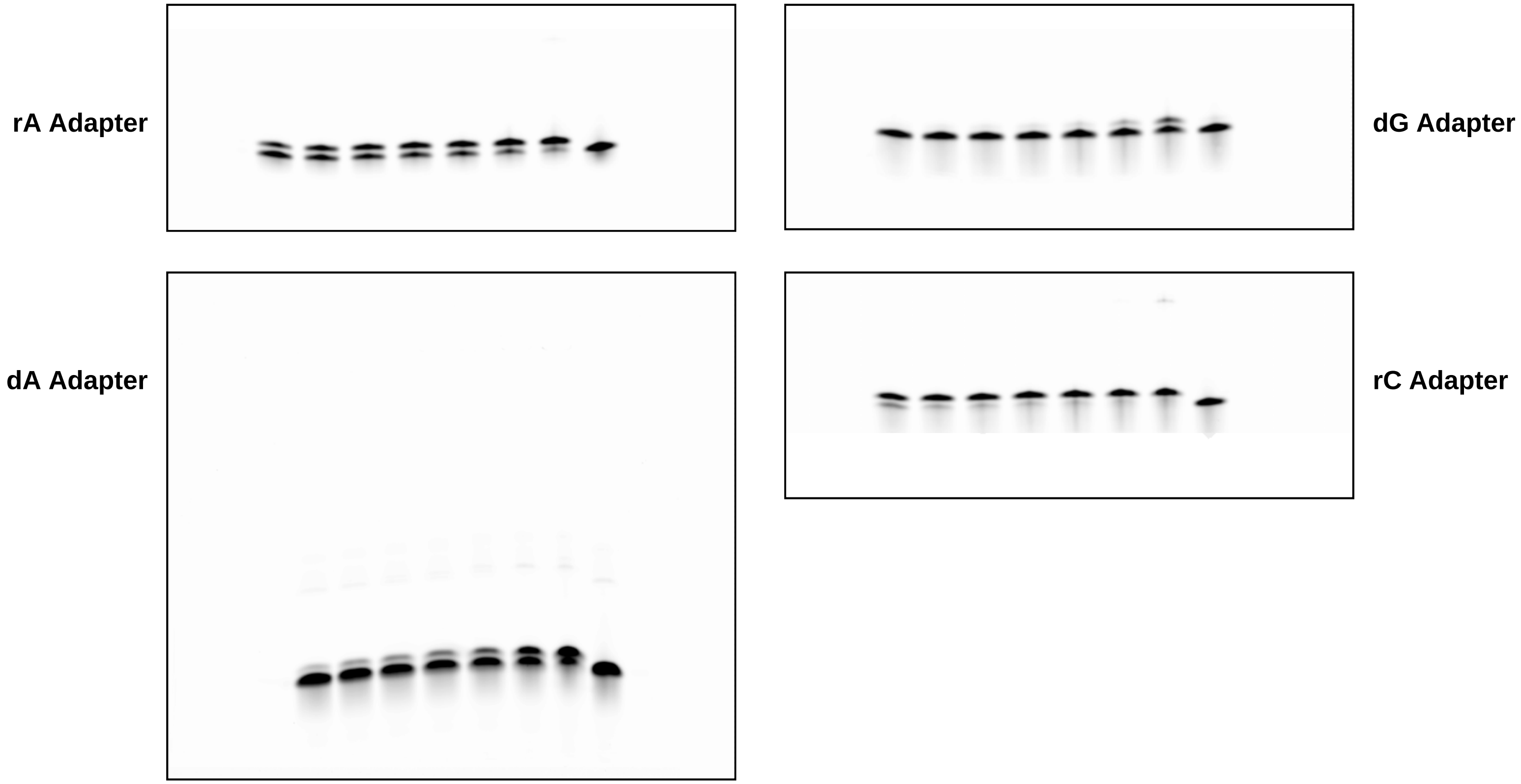

Supplemental Figure S2. Uncropped gel images of Figure 1c.

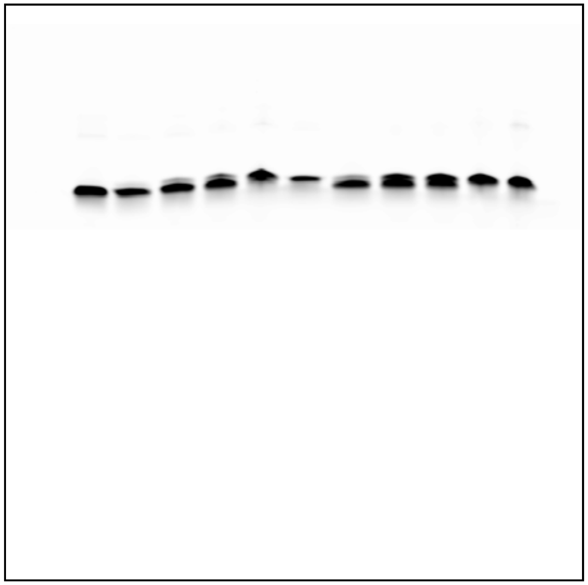

Supplemental Figure S3. Uncropped gel image of Figure 1d

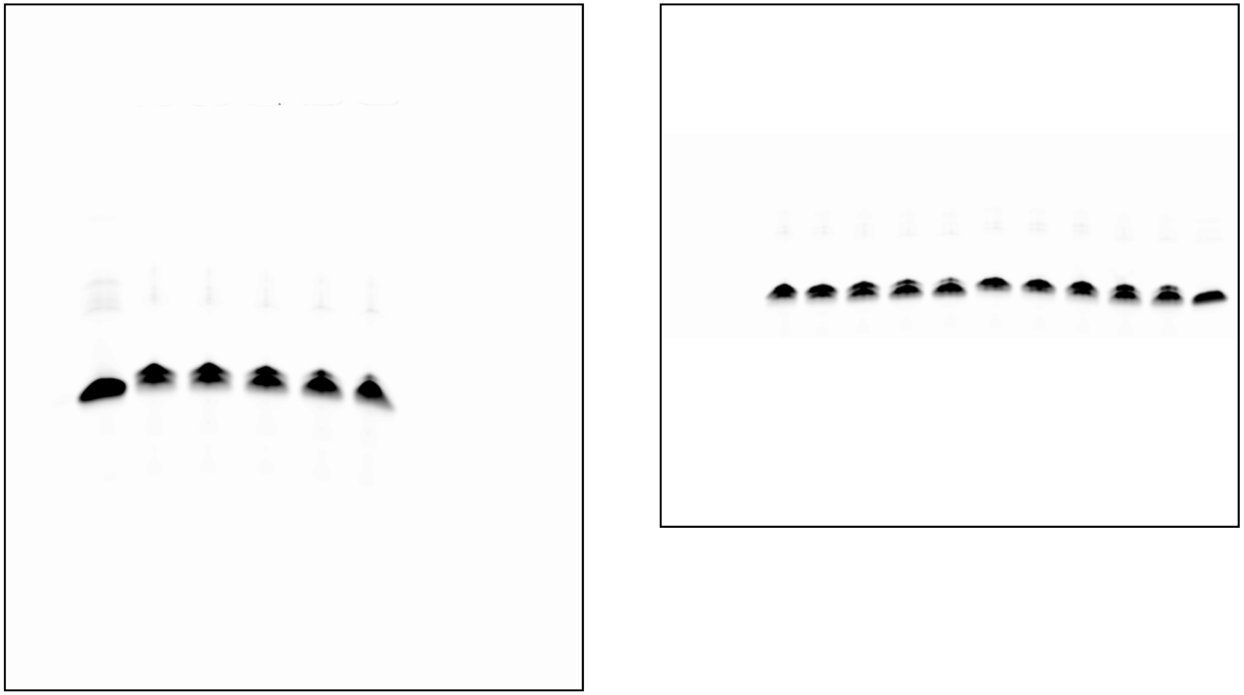

Supplemental Figure S4. Uncropped gel images of Figure 2a

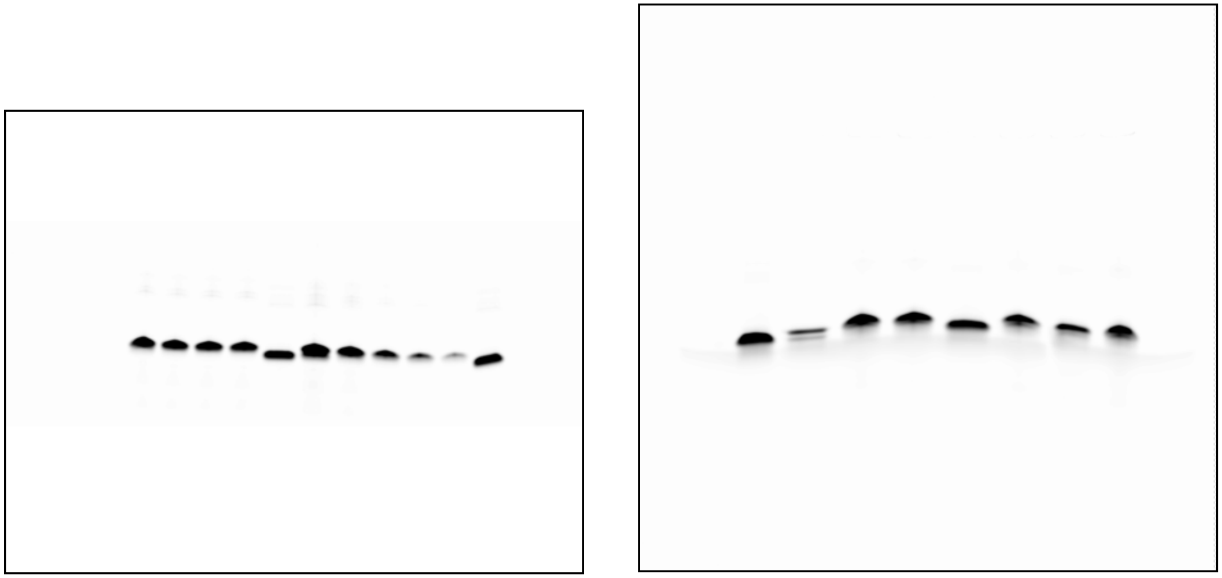

Supplemental Figure S5. Uncropped gel images of Figure 2b and Figure 2d.

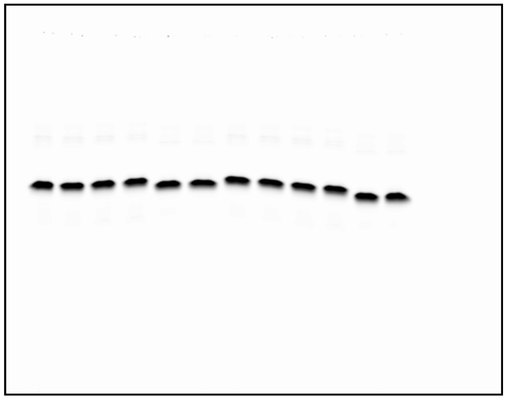

Supplemental Figure S6. Uncropped gel image of Figure 2c

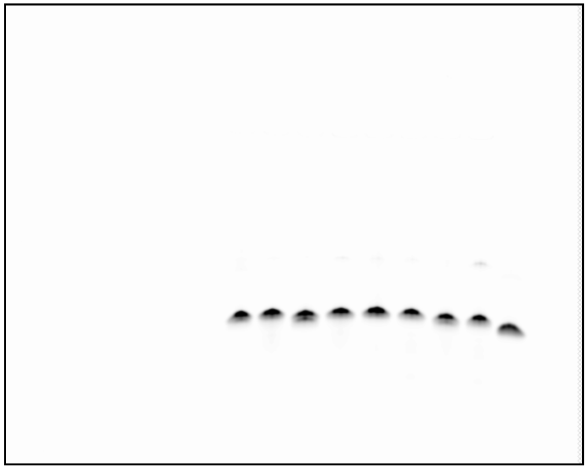

Supplemental Figure S7. Uncropped gel image of Figure 3

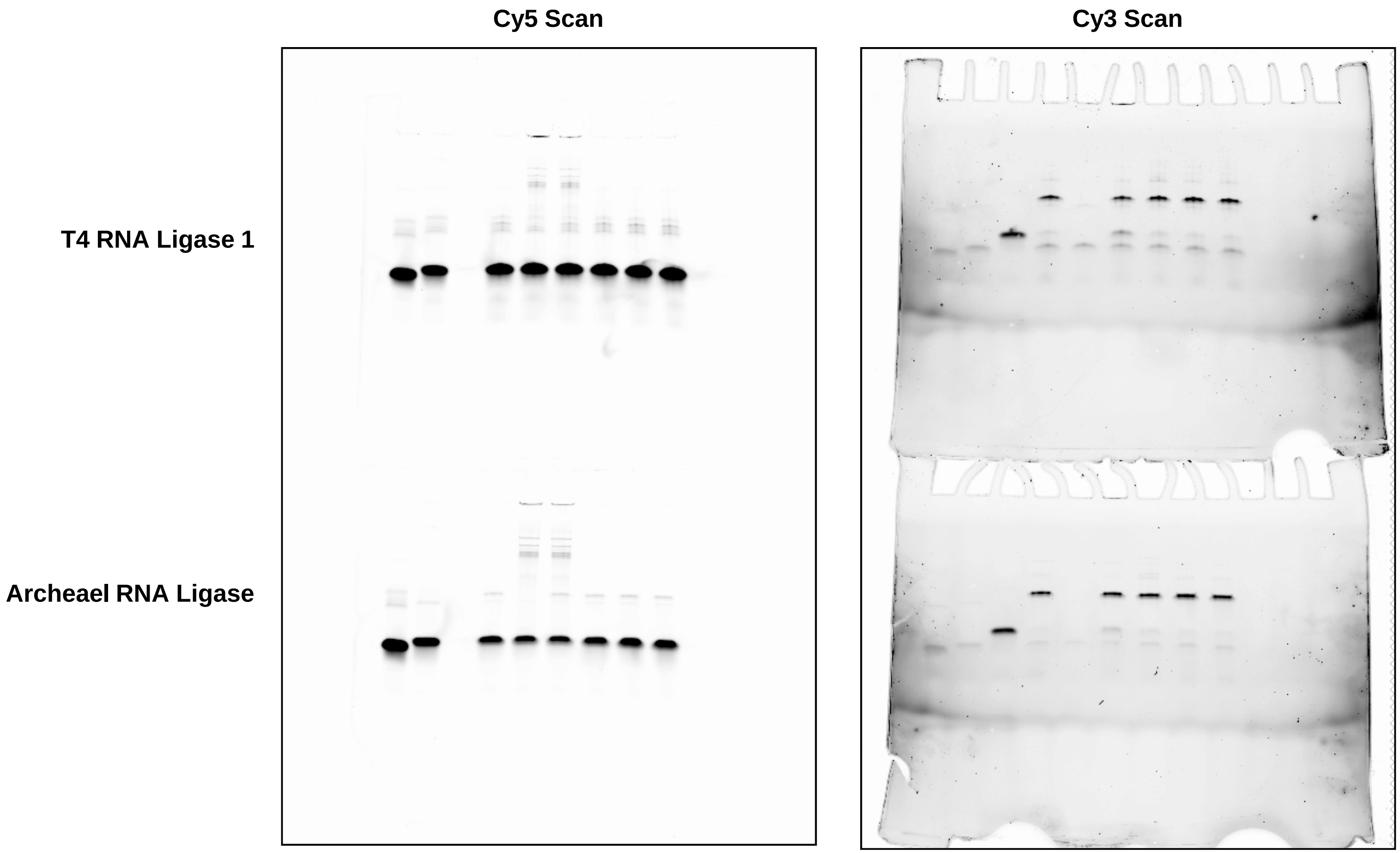

Supplemental Figure S8. Uncropped gel image of Figure 4a and Figure 4c

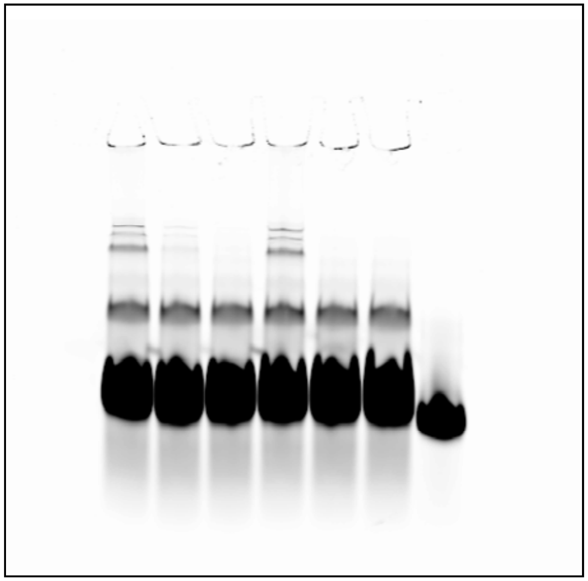

Supplemental Figure S9. Uncropped gel image of Figure 4b

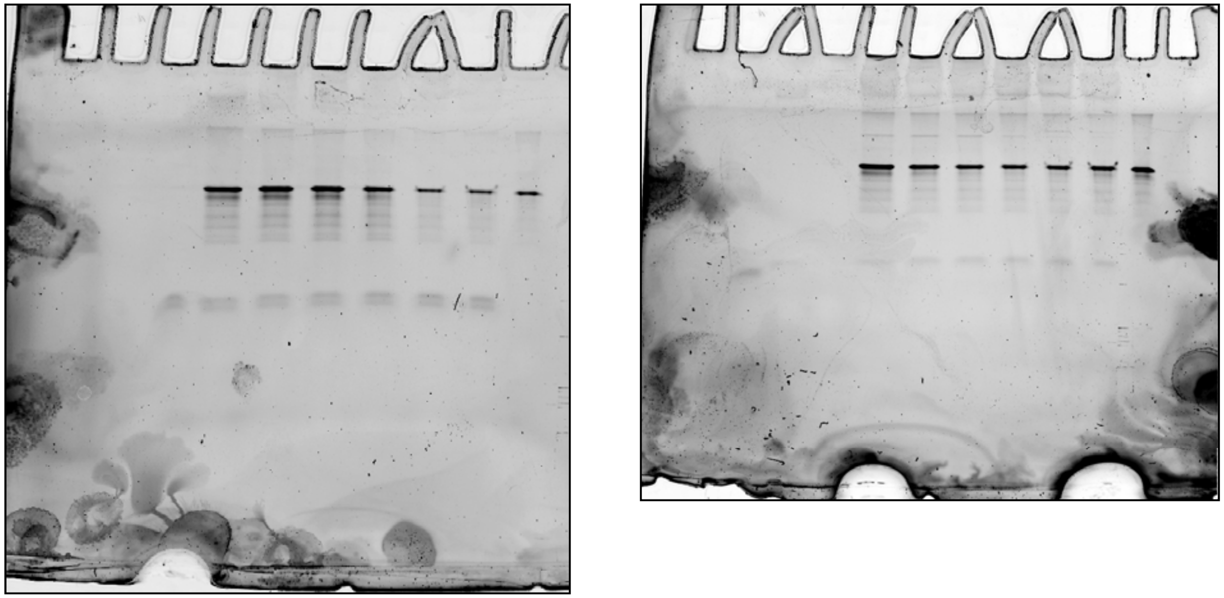

Supplemental Figure S10. Uncropped gel image of Figure 5
